# The Potential Roles of Ghrelin in Metabolic Syndrome and Secondary Symptoms of Alzheimer’s Disease

**DOI:** 10.3389/fnins.2020.583097

**Published:** 2020-09-24

**Authors:** Sujin Kim, Yunkwon Nam, Soo Jung Shin, Yong Ho Park, Seong Gak Jeon, Jin-il Kim, Min-Jeong Kim, Minho Moon

**Affiliations:** ^1^Department of Biochemistry, College of Medicine, Konyang University, Daejeon, South Korea; ^2^Department of Neural Development and Disease, Korea Brain Research Institute (KBRI), Daegu, South Korea; ^3^Department of Nursing, College of Nursing, Jeju National University, Jeju-si, South Korea

**Keywords:** ghrelin, Alzheimer’s disease, metabolic syndrome, depression, sleep–wake disturbances, abnormal eating behaviors

## Abstract

Although the major causative factors of Alzheimer’s disease (AD) are the accumulation of amyloid β and hyperphosphorylated tau, AD can also be caused by metabolic dysfunction. The major clinical symptom of AD is cognitive dysfunction. However, AD is also accompanied by various secondary symptoms such as depression, sleep–wake disturbances, and abnormal eating behaviors. Interestingly, the orexigenic hormone ghrelin has been suggested to have beneficial effects on AD-related metabolic syndrome and secondary symptoms. Ghrelin improves lipid distribution and alters insulin sensitivity, effects that are hypothesized to delay the progression of AD. Furthermore, ghrelin can relieve depression by enhancing the secretion of hormones such as serotonin, noradrenaline, and orexin. Moreover, ghrelin can upregulate the expression of neurotrophic factors such as brain-derived neurotrophic factor and modulate the release of proinflammatory cytokines such as tumor necrosis factor α and interleukin 1β. Ghrelin alleviates sleep–wake disturbances by increasing the levels of melatonin, melanin-concentrating hormone. Ghrelin reduces the risk of abnormal eating behaviors by increasing neuropeptide Y and γ-aminobutyric acid. In addition, ghrelin increases food intake by inhibiting fatty acid biosynthesis. However, despite the numerous studies on the role of ghrelin in the AD-related pathology and metabolic disorders, there are only a few studies that investigate the effects of ghrelin on secondary symptoms associated with AD. In this mini review, our purpose is to provide the insights of future study by organizing the previous studies for the role of ghrelin in AD-related pathology and metabolic disorders.

## Introduction

Alzheimer’s disease (AD), characterized histopathologically by amyloid β aggregation and tau hyperphosphorylation, is the most common cause of dementia (Querfurth and LaFerla, 2010). Although AD is clinically characterized by progressive impairment of cognitive functions such as episodic memory, it is also accompanied by secondary symptoms such as depression, sleep–wake disturbances, and abnormal eating behaviors. Notably, some AD patients exhibit symptoms of major depressive episodes such as appetite changes, insomnia, and dysphoria (Merriam et al., 1988; Novais and Starkstein, 2015; Okuda et al., 2019). In addition, subjects with mild cognitive or behavioral impairment are more likely to experience accelerated progression to AD or onset of dementia if they have a history of depression (Wilson et al., 2002). Furthermore, metabolic syndrome such as hyperglycemia, hyperinsulinemia, and hypercholesterolemia is known to be the risk factor for AD (Lane and Farlow, 2005; Nelson and Alkon, 2005; Razay et al., 2007). Psychiatric and metabolic deficits are not only symptoms of AD, but also markers of AD prognosis. Although there are drugs that effectively delay AD-related cognitive impairment, thus far, no therapeutic strategy has been established to treat the psychiatric and metabolic symptoms of AD thus far.

Ghrelin is an orexigenic hormone which regulates body weight, energy homeostasis, and metabolism through the hypothalamus, and plays an enhancing role in insulin resistance and growth hormone secretion (Pradhan et al., 2013; Muller et al., 2015; Yanagi et al., 2018). Remarkably, extensive evidence has indicated that ghrelin may alleviate AD-related pathology such as Aβ accumulation (Dhurandhar et al., 2013; Jeong et al., 2018), tau hyperphosphorylation (Kang et al., 2015), mitochondrial dysfunction (Chung et al., 2007), impaired adult neurogenesis (Moon et al., 2014), and neuroinflammation (Moon et al., 2011; Sibilia et al., 2012). Therefore, due to its potential for mitigating AD-related pathologies, ghrelin could be a possible therapeutic target for AD (Jeon et al., 2019). In addition, several studies have reported that ghrelin plays a protective role in metabolic syndrome (Broglio et al., 2004) and various psychiatric disorders, including depression (Carlini et al., 2012), sleep–wake disturbances (Yannielli et al., 2007), and abnormal eating behaviors (Overduin et al., 2012). However, the possible roles of ghrelin in AD-related metabolic syndrome and psychiatric disorders have not yet been investigated. Furthermore, although ghrelin plays a pivotal role in energy metabolism and homeostasis (Yanagi et al., 2018), the effects of ghrelin on metabolic disorders and secondary symptoms of AD remain unclear. In this review, we discuss the possibility of using ghrelin as a therapeutic target for AD by presenting evidence for the potential roles of ghrelin in the metabolic symptoms and secondary symptoms associated with AD.

## The Role of Ghrelin in Metabolic Syndrome and Secondary Symptoms of AD

### The Role of Ghrelin in AD-Related Metabolic Syndrome

Alzheimer disease is considered to be another type of diabetes, and hyperinsulinemia and hypercholesterolemia are known to promote AD pathogenesis (de La Monte and Wands, 2008; Merlo et al., 2010). Hyperinsulinemia inhibits the activity of AMP-activated protein kinase (AMPK) (Valentine et al., 2014), and inhibition of AMPK activity as a result of metabolic syndrome inactivates the pentose phosphate pathway (Saito et al., 2015). Abnormal metabolic conditions including diabetes mellitus may induce impairment of energy metabolism by increasing the production of reactive oxygen species and mitochondrial dysfunction (Bonomini et al., 2015; Bhatti et al., 2017) and may accelerate cognitive impairment by promoting abnormal release of neurotransmitters, particularly γ-aminobutyric acid (GABA) (van Bussel et al., 2016). Several studies have suggested that in neurodegenerative diseases, there exists a link between insulin and cholesterol levels (Laws et al., 1991; Nelson and Alkon, 2005). Indeed, insulin increases the activity of 3-hydroxy-3-methylglutaryl-CoA reductase, the enzyme that catalyzes an intermediate in cholesterol synthesis (Nelson and Alkon, 2005). In a previous study, individuals with type 2 diabetes mellitus exhibited decreased cholesterol absorption and increased cholesterol synthesis regardless of obesity (Simonen et al., 2002). In the case of AD, Aβ-induced metabolic imbalance involving AMPK results in tau phosphorylation and neuroinflammation (Martinez de Morentin et al., 2010; Thornton et al., 2011; Lee et al., 2013). Furthermore, AD patients suffer from insulin signaling dysfunction due to a reduction in activity of tyrosine kinase, an important effector system for insulin receptors (Frolich et al., 1999), and decreased activities of elements of insulin–PI3K–AKT signaling, which results in elevated tau phosphorylation and decreased glucose metabolism (Liu et al., 2011). In particular, apolipoprotein E (ApoE), a protein responsible for the metabolism of plasma lipids (Jones et al., 2019), is also associated with AD (Lane and Farlow, 2005). Reportedly, polymorphism of ApoE allele, especially ApoE ε4, attributes to risk of AD development by increasing Aβ and Tau aggregation, whereas ApoE ε2 exhibits protective effects on risk of AD development (Verghese et al., 2011). Moreover, ApoE ε4 induces dysregulation of cerebral metabolism by decreasing lipid and glucose metabolism (Brandon et al., 2018). Interestingly, the control of insulin and plasma glucose by ghrelin administration can vary depending on the details of administration (i.e., duration, route, and dose) (Nieminen and Mustonen, 2004; Theander-Carrillo et al., 2006; Barazzoni et al., 2007a; Goshadrou et al., 2015). In rats, acute (1 day) administration of ghrelin increased levels of insulin and fasting plasma glucose, but chronic (21 days) administration of ghrelin normalized these upregulations (Goshadrou et al., 2015). The mechanism of insulin and glucose regulation after administration of exogenous ghrelin has not yet been clearly identified. Known mechanisms through which insulin inhibits ghrelin include upregulation of the AMPK- uncoupling protein 2 (UCP2) pathway through AMPK phosphorylation and UCP2 expression (Chmielewska et al., 2010; Wang et al., 2010), and the IA-2β pathway, which inhibits glucose-stimulated insulin through induction of IA-2β (Doi et al., 2006). These two pathways independently inhibit insulin. Remarkably, ghrelin not only regulates insulin but also regulates nigrostriatal dopamine function in a UCP2-dependent manner (Andrews et al., 2009). In addition, upregulation of UCP2 has been demonstrated to have a protective effect in animal models of ischemic stroke and Parkinson disease (Andrews et al., 2009; Liu et al., 2009).

The concentration of ghrelin is decreased in the middle-aged and elderly people with metabolic syndrome compared to individuals of the same age who do not have metabolic syndrome, and its concentration rapidly is decreased as metabolic abnormalities intensify (Ukkola et al., 2006; Serra-Prat et al., 2009; Mora et al., 2014). Several studies have suggested that ghrelin may be involved in the metabolism of insulin and glucose. In healthy subjects, administration of acyl-ghrelin reduced insulin levels and increased glucose levels (Broglio et al., 2004). By contrast, administration of des-acyl-ghrelin improved glucose metabolism and insulin sensitivity in subjects (Benso et al., 2012). In addition, administration of acyl-ghrelin alone to growth hormone–deficient patients increases insulin and glucose levels rapidly but decreases insulin sensitivity, whereas administration of acyl-ghrelin and des-acyl-ghrelin increases insulin sensitivity (Gauna et al., 2004). Moreover, transgenic mice overexpressing des-acyl-ghrelin exhibited a reduction in white adipose tissue weight and improvement in glucose tolerance and insulin sensitivity (Zhang et al., 2008). In a previous study, obese children with metabolic syndrome exhibited decreased levels of des-acyl-ghrelin and an increased acyl-ghrelin/des-acyl-ghrelin ratio compared to obese children without metabolic syndrome (Pacifico et al., 2009). Similarly, obese individuals with normoglycemia and type 2 diabetes mellitus exhibited increased plasma levels of acyl-ghrelin and decreased levels of des-acyl-ghrelin compared to lean individuals (Rodriguez et al., 2009). Therefore, individuals with metabolic syndrome and obesity have a higher acyl-ghrelin/des-acyl-ghrelin ratio than non-obese individuals with metabolic syndrome, suggesting that excessive acyl-ghrelin levels may promote insulin resistance (Barazzoni et al., 2007b). Moreover, administration of ghrelin causes tissue-specific changes in the activity of mitochondrial oxidative enzyme, the expression of gene involved in lipid metabolism, and triglyceride content in rats, suggesting that ghrelin may be involved in the regulation of lipid distribution and metabolism (Barazzoni et al., 2005).

Patients with AD exhibited lower lean mass compared to controls. Although patients with AD and controls exhibited similar basal levels of ghrelin, the area under the curve value was lower in male patients with AD than in control males (Theodoropoulou et al., 2012). Although further evidence and investigation are required, a previous study by Yoshino et al. (2018) showed increased levels of serum acyl-ghrelin in AD subjects compared to control subjects that might be a result of changes of the ghrelin pathway in brain (Yoshino et al., 2018). Thus, further deliberate examination and interpretation should be made. Given that ghrelin-O-acyltransferase blockade reduces the acyl-ghrelin/des-acyl-ghrelin ratio, des-acyl-ghrelin administration could be a promising therapeutic approach for metabolic dysfunction (Barnett et al., 2010). It is possible that the increased acyl-ghrelin/des-acyl-ghrelin ratio in individuals with obesity may promote insulin resistance and hyperinsulinemia (Barazzoni et al., 2007b). Insulin resistance and hyperinsulinism may increase the prevalence of AD by increasing Aβ-related metabolism and inflammation in the brain (Craft, 2007). Additionally, insulin transport to the brain is reduced, causing insulin deficiency (Baura et al., 1996). Furthermore, neurofibrillary tangles containing phosphorylated tau were observed in the hippocampus of insulin receptor substrate 2 knockout mice, indicating that insulinlike growth factor-1 and insulin are associated with tau phosphorylation (Schubert et al., 2003). These results suggest that metabolic abnormalities such as hyperinsulinemia and insulin resistance promote AD development (DiStefano et al., 2007). Both *in vitro* and *in vivo* studies have reported that an optimal concentration of insulin reduced Aβ production through increasing the levels of α-secretase ADAM10, sAPPα, and C83 and decreasing the levels of β-secretase BACE1, sAPPβ, and C99 (Vandal et al., 2014; Wang et al., 2014). Furthermore, antidiabetic drugs such as metformin and peroxisome proliferator-activated receptor-γ agonists may have beneficial effects on preventing or improving cognitive dysfunction and pathogenesis of AD (Crisby et al., 2002; Cong et al., 2010; Akter et al., 2011). Therefore, given that ghrelin plays major roles in metabolism, it may be a noteworthy therapeutic target for AD (Gahete et al., 2011; Eslami et al., 2018). Nonetheless, considering the fact that the area under the curve value of ghrelin was increased by glucose loading only in male patients with AD, not in female patients (Theodoropoulou et al., 2012), and the higher basal ghrelin levels in female healthy and opposite-sex twin pair subjects than men (Makovey et al., 2007; Song et al., 2017), difference in effects of ghrelin for AD-related metabolic syndrome according to gender should be examined in the future.

### The Role of Ghrelin in AD-Related Depression

Depression is the most common secondary symptom in patients with AD and is associated with accelerated cognitive impairment (Bassuk et al., 1998; Modrego, 2010). In particular, late-onset depression is considered to be a risk factor for AD development and is more strongly associated with cognitive decline than early-onset depression (Devanand et al., 1996; van Reekum et al., 1999; Wilson et al., 2002). An increase in glucocorticoid production is characteristic of early AD (Rasmuson et al., 2001), and hypothalamic–pituitary–adrenal (HPA) axis dysfunction caused by excessive glucocorticoid secretion and reactivity promotes the development of depression (Zunszain et al., 2011). In addition, the limbic lobe, hippocampus, amygdala, and anterior and posterior cingulate cortices are involved in the pathophysiology of depression; a decrease in the density/structural plasticity of these areas has been identified in patients with depression (Rajkowska, 2000; Nestler et al., 2002; Ries et al., 2009) and in patients with early AD (Braak et al., 1993; Minoshima et al., 1997; Gastard et al., 2003; Poulin et al., 2011). Moreover, dysfunction of the monoaminergic system, in particular the serotonergic and noradrenergic systems, has been shown to occur in both depression and AD (Ressler and Nemeroff, 2000; Versijpt et al., 2003; Kepe et al., 2006; Chalermpalanupap et al., 2013).

Chronic stress–induced glucocorticoid upregulation promotes neuronal damage, induces structural changes, and decreases the expression of brain-derived neurotrophin-3 and neurotrophic factor (BDNF) mRNA in the hippocampus (Smith et al., 1995; Nestler et al., 2002). Ghrelin, which has a protective effect on metabolic disturbances induced by chronic stress, has been reported to also have protective effects against depressive-like responses in experimental animals (Lutter et al., 2008; Labarthe et al., 2014). In addition, the rat model of diabetes exhibits lower hippocampal BDNF mRNA levels compared to control rats, while administration of ghrelin significantly upregulates BDNF mRNA levels in a rat model of diabetes (Ma et al., 2011). Olfactory bulbectomy induced depressive-like behavior in mice, and this deficit was reversed by ghrelin administration, indicating that ghrelin exhibits an antidepressant-like effect (Carlini et al., 2012). Moreover, olfactory bulbectomy decreased noradrenaline levels and serotonin turnover and increased the levels of proinflammatory cytokines such as interleukin 1β (IL-1β) and tumor necrosis factor α (TNF-α) (Hellweg et al., 2007; Song et al., 2009; Yang et al., 2014; Chang et al., 2016). However, exogenous ghrelin inhibited the release of proinflammatory cytokines and increased noradrenaline levels and serotonin turnover, further demonstrating the antidepressant-like effect of ghrelin (Date et al., 2006; Kawakami et al., 2008; Waseem et al., 2008; Hansson et al., 2014). Moreover, increased ghrelin levels induced by calorie restriction led to anti–depressant-like effects. By contrast, the calorie restriction–induced anti–depressive-like effects were not observed in growth hormone secretagogue receptor (GHS-R) null mice, and these animals exhibited increased social avoidance compared to their wild-type littermates (Lutter et al., 2008). Notably, GHS-R1 is known to be involved in various psychological conditions, including depression (Guo et al., 2019). Thus, ghrelin may alleviate depressive-like responses by acting on GHS-R1–expressing neurons (Abizaid et al., 2006; Diano et al., 2006; Lutter et al., 2008).

Mechanisms related to the pathogenesis of depression include HPA axis dysfunction, monoaminergic system deficiency, inflammation, and neurodegeneration (Zunszain et al., 2011). Therefore, ghrelin may alleviate depressive symptoms by upregulating BDNF mRNA, decreasing glucocorticoid levels, rebalancing the monoaminergic system, stimulating GHS-R1–expressing neurons to modulate mood and synapse formation, and regulating the release of proinflammatory cytokines such as IL-1β and TNF-α. Unfortunately, few studies have investigated the role of ghrelin in AD-related depression. However, given the antidepressant-like effect of ghrelin observed in previous animal studies, we hypothesize that ghrelin may have a therapeutic effect on depression in AD patients.

Other neuropeptides, including neurotensin and neuropeptide Y (NPY), have been shown to be involved in the pathogenesis of depression. Interestingly, the effects of neurotensin were opposite to those of ghrelin on food intake (Cooke et al., 2009). In addition, neurotensin neurons are known to play important roles in regulation of energy balance controlled by ghrelin and leptin (Brown et al., 2017). Notably, mRNA levels of ghrelin and expression of its G protein–coupled receptors (neurotensin receptors 1 and 2) are decreased, whereas levels of neurotensin tend to decrease in the temporal lobe of patients with AD (Gahete et al., 2010). In another study, density of amyloid plaque in the occipital cortex was negatively correlated with density of neurotensin neurons in postmortem suprachiasmatic nucleus (SCN) (Hu et al., 2013). Moreover, neurotensin receptor 1 knockout mice showed increased depressive-like behaviors in the tail suspension test (Fitzpatrick et al., 2012). Despite the conflicting results from clinical studies examining the roles of NPY in depression, evidence strongly supports the involvement of NPY in pathogenesis of depression (Morales-Medina et al., 2010). In addition, levels of NPY vary by the locations of sampling and models of AD (Duarte-Neves et al., 2016). Considering, ghrelin cross talks with NPY neurons in the arcuate nucleus (ARC) in rats (Kohno et al., 2003) and the evidence that ghrelin increases gene expression of NPY in the ARC in hypothalamic cultures of rats (Goto et al., 2006), the regulatory effect of ghrelin on NPY in AD-related depression should be examined in the future. Although the interacting mechanisms among ghrelin, neurotensin, and NPY in AD-related depression remain to be examined, neurotensin and NPY, at least, seem to be mediating some AD-related depression-like behaviors by interacting with ghrelin.

### The Role of Ghrelin in AD-Related Sleep–Wake Disturbances

Maintaining a normal circadian rhythm is essential in order to optimize quality of life and preserve health. Sleep–wake disturbances are common secondary symptoms of AD that have been observed in studies on patients with AD (Uddin et al., 2020) and on the 3×Tg and 5×FAD mouse models of AD (Sterniczuk et al., 2010; Sethi et al., 2015). Moreover, the pineal gland, which adjusts sleep patterns by producing melatonin, and the SCN, which is involved in the regulation and production of biological rhythms, are vulnerable regions in AD (Buijs and Kalsbeek, 2001; Wu and Swaab, 2005; Roy et al., 2019). A recent study using magnetic resonance imaging of the brain showed that the pineal volume was decreased in mild cognitive impairment (MCI) patients who converted to AD than in MCI patients who did not convert to AD (Matsuoka et al., 2020). Furthermore, sleep–wake cycle disturbances showed to increase Aβ plaques in the brain of AD mouse models (Kang et al., 2009; Rothman et al., 2013). The level of Aβ_42_ protein in cerebrospinal fluid of healthy middle-aged individuals was increased in the sleep deprivation group compared to that in the unrestricted sleep group (Ooms et al., 2014). In particular, sundowning, a common symptom of AD with circadian rhythm disruption, occurs in the afternoon and evening and is accompanied by seven destructive actions: combativeness, agitation or purposeless movement, wandering, prolonged incoherent vocalization, hallucinations, confusion, and disorientation (Gallagher-Thompson et al., 1992; Volicer et al., 2001). Regulations of sleep and brain functions are related to regulatory pathways including hippocampal signaling pathway and common neurotransmitter systems such as orexinergic and GABAergic systems (Prince and Abel, 2013). However, dysfunction of sleep function destabilizes physiology, disturbs sleep–wake timing, and promotes other pathological symptoms such as cognitive and metabolic deficits (Wulff et al., 2010). Surprisingly, the orexigenic peptide ghrelin regulates circadian rhythm (Yannielli et al., 2007; LeSauter et al., 2009; Steiger et al., 2011). Studies have been shown that administration of ghrelin decreased REM sleep and increased slow wave sleep in elderly men (Kluge et al., 2010) and promoted non-REM sleep in male mice (Obal et al., 2003). Several studies have been reported that GHS-R1 mRNA is highly expressed in the SCN (Zigman et al., 2006) and ARC (Jeon et al., 2019). It is well known that neurons in the SCN are projected to the dorsal parvocellular paraventricular nucleus (PVHd), and neurons in the PVHd are projected to sympathetic preganglionic neurons, which in turn regulate melatonin secretion by the pineal gland (Saper et al., 2005). Therefore, ghrelin could alleviate sleep–wake disturbances through increasing melatonin secretion by binding to GHS-R1 in the SCN and enhancing the regulatory pathways that stimulate the pineal gland. Additionally, the ARC neurons innervate to the ventrolateral preoptic nucleus (VLPO) and lateral hypothalamus (LH) via the dorsomedial hypothalamus. The VLPO is involved in sleep, and the LH is associated with wakefulness by regulating melanin-concentrating hormone (Saper et al., 2005). Thus, ghrelin could enhance sleep–wake cycle by stimulating the VLPO and LH through binding to GHS-R1 in the ARC. Moreover, ghrelin affects circadian locomotor output cycles kaput (CLOCK)–dependent functions (Garaulet et al., 2011). Taken together, these data indicate that ghrelin may alleviate sleep-wake disturbances by stimulating the SCN and ARC and ultimately regulate the function of CLOCK-related activity.

Areas such as the SCN, ARC, and pineal gland that influence the regulation and production of biological rhythms are damaged in patients and mouse models of AD, and these damaged regions cause sleep–wake disturbances (Do et al., 2018; Roy et al., 2019; Matsuoka et al., 2020). In addition, sleep–wake disturbances increase level of Aβ protein and plaques in healthy individuals and mouse models of AD (Kang et al., 2009; Rothman et al., 2013). Accumulating evidence has demonstrated that ghrelin not only has beneficial effects on sleep–wake cycle, but also stimulates areas involved in biological rhythms (Yannielli et al., 2007; LeSauter et al., 2009; Steiger et al., 2011). Because there is almost no study on the effects of ghrelin in AD-related sleep–wake disorders, further well-controlled clinical trials regarding the positive effects of ghrelin on disruption in the circadian rhythm and quality of life in patients with AD are needed.

### The Role of Ghrelin in AD-Related Abnormal Eating Behaviors

In a previous clinical study, patients with AD exhibited weight loss (Barrett-Connor et al., 1998). Aging causes changes in appetite and growth hormone secretion (Creyghton et al., 2004), and these changes are referred to as “anorexia of aging.” Anorexia of aging causes several sequelae such as undernutrition, frailty, and sarcopenia (Cox et al., 2019). In addition, aging increases insulin resistance and reduces glucose metabolism (Shou et al., 2020). Insulin levels increase with age, and insulin may promote the development of anorexia. Moreover, aging-related leptin and ghrelin resistance may be related to anorexia of aging (Chapman et al., 2002; Chapman, 2004, 2007; Di Francesco et al., 2007).

Ghrelin is known to increase food intake (Tschop et al., 2000; Wren et al., 2000; Wren et al., 2001) and promote gastric emptying (Inui et al., 2004; Overduin et al., 2012). In particular, ghrelin regulates fatty acid metabolism in the ventromedial nuclei of the hypothalamus (VMH) to regulate food intake. The orexigenic effect of ghrelin is mediated via the phosphorylation of hypothalamic AMPK, which decreases malonyl-CoA levels and increases carnitine palmitoyltransferase-1 activity (Lopez et al., 2008). In the hypothalamic ARC, agouti-related protein and NPY are expressed in orexigenic neurons, and proopiomelanocortin (POMC) and amphetamine- and cocaine-regulated transcript are expressed in anorexigenic neurons (Zheng et al., 2003; Chen et al., 2004). In a previous study, ghrelin suppressed the activity of POMC-expressing neurons in the ARC by activating NPY-expressing neurons, which promoted the release of GABA (Cowley et al., 2003). Moreover, ghrelin reduced malonyl-CoA levels by suppressing the expression of fatty acid synthase in the VMH (Lopez et al., 2008). Indeed, intracerebroventricular infusion of ghrelin stimulated food intake via a mechanism involving the dopamine D_1_ receptor in rats (Overduin et al., 2012). In addition, ghrelin administration stimulated cerebral responses to food in the amygdala, anterior insula, orbitofrontal cortex, and striatum of healthy subjects (Malik et al., 2008). Interestingly, rivastigmine administration increased appetite by increasing acyl-ghrelin/des-acyl-ghrelin ratio in AD patients (Furiya et al., 2018) implying AD-related cachexia could potentially be alleviated by promoting appetite through ghrelin administration. Thus, the orexigenic effect of ghrelin may prevent the loss of body weight and lean mass in AD patients.

## Conclusion

**FIGURE 1:**
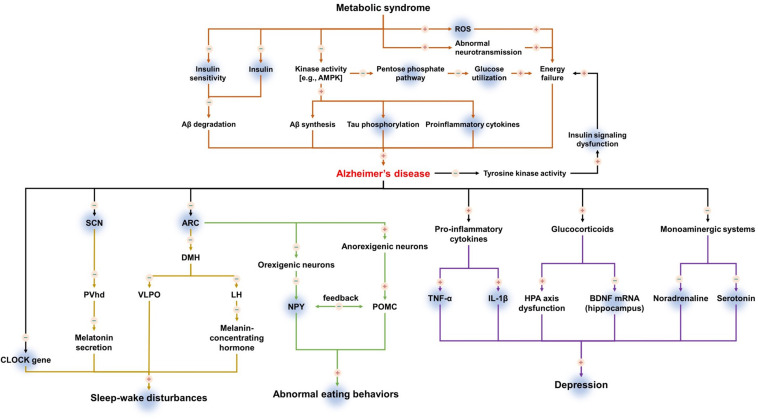
The mechanism of action of ghrelin in metabolic syndrome and secondary symptoms of Alzheimer’s disease. Upregulation is indicated by plus (+), downregulation is indicated by minus (–). Shadowed background color indicates the beneficial effects of ghrelin on metabolic syndrome and secondary symptoms of Alzheimer’s disease.

Taken together, there has been a lack of evidence demonstrating that ghrelin can alleviate metabolic syndrome and secondary symptoms associated with AD. However, it has been suggested that ghrelin may affect the progression of AD by alleviating metabolic syndrome. Moreover, it is thought that ghrelin may control secondary symptoms of AD such as depression, sleep–wake disturbances, and abnormal eating behaviors (Table 1 and Figure 1). Given the evidence for the involvement of ghrelin at various stages of AD progression, it is necessary to further examine the role of ghrelin in metabolic syndrome and in the secondary symptoms of AD.

**TABLE 1:** The role of ghrelin in metabolic syndrome and secondary symptoms of Alzheimer’s disease.

	Subjects or experimental models	Major findings	References
Metabolic syndrome	Growth hormone–deficient patients	Combined treatment with acyl-ghrelin and des-acyl-ghrelin enhanced insulin sensitivity, while administration of acyl-ghrelin alone reduced insulin sensitivity	Gauna et al., 2004
	Patients with metabolic syndrome	Patients with metabolic syndrome exhibited lower total ghrelin levels and a higher acyl-ghrelin/des-acyl-ghrelin ratio than non-obese individuals with metabolic syndrome	Barazzoni et al., 2007b
	Obese children with metabolic syndrome	Obese children with metabolic syndrome exhibited decreased levels of des-acyl-ghrelin and an increased acyl-ghrelin/des-acyl-ghrelin ratio compared to obese children without metabolic syndrome	Pacifico et al., 2009
	Obese patients with normoglycemia and type 2 diabetes mellitus	Obese individuals with normoglycemia and type 2 diabetes mellitus exhibited increased plasma levels of acyl-ghrelin and decreased levels of des-acyl-ghrelin compared to lean individuals	Rodriguez et al., 2009
	Patients with moderate Alzheimer’s disease	Patients with Alzheimer’s disease exhibited a lower area under the curve value for ghrelin compared to control patients	Theodoropoulou et al., 2012
	Healthy young male subjects	Acyl-ghrelin reduced insulin levels and increased glucose levels, whereas des-acyl-ghrelin antagonized these effects	Broglio et al., 2004
	Healthy young subjects	Administration of des-acyl-ghrelin reduced the area under the curve for glucose and free fatty acid. In addition, des-acyl-ghrelin time-dependently increased the area under the curve of insulin	Benso et al., 2012
	Isolated rat adipocytes	Acyl-ghrelin inhibited lipolysis	Muccioli et al., 2004
	Isolated mice pancreatic islets	Acyl-ghrelin decreased spontaneous pancreatic polypeptide release, and des-acyl-ghrelin counteracted this	Kumar et al., 2010
	ddY mice	Administration of des-acyl-ghrelin decreased food intake and gastric emptying and increased the gene expression of hypothalamic neuropeptides such as cocaine- and amphetamine-regulated transcript and urocortin	Asakawa et al., 2005
	Transgenic mice overexpressing des-acyl-ghrelin	Mice overexpressing des-acyl-ghrelin exhibited a decrease in gastric emptying rate, body weight, food intake, fat pad mass, and plasma triglyceride levels	
	Transgenic mice overexpressing des-acyl-ghrelin	Overexpression of des-acyl-ghrelin inhibited adipose tissue development and improved glucose tolerance and insulin sensitivity	Zhang et al., 2008
	C57BL/6 mice	Inhibition of ghrelin-O-acyltransferase reduced body weight and fat mass	Barnett et al., 2010
Depression	Mice subjected to bilateral olfactory bulbectomy	Intracerebroventricular administration of ghrelin reversed the depressive-like phenotype induced by olfactory bulbectomy	Carlini et al., 2012
	Calorie-restricted mice growth hormone secretagogue receptor null mice	Increased ghrelin levels induced by calorie restriction promoted antidepressant-like responses, whereas these effects were abolished in growth hormone secretagogue receptor null mice	Lutter et al., 2008
Sleep–wake disturbances	Sprague–Dawley rats	Microinjection of ghrelin into the lateral hypothalamus stimulated wakefulness and food consumption	Szentirmai et al., 2007
	C57BL/6J mice mPeriod2^Luciferase^ mice	After food deprivation, intraperitoneal injection of ghrelin or growth hormone–releasing peptide-6 altered circadian rhythm by directly acting on the suprachiasmatic nucleus	Yannielli et al., 2007
	Overweight/obese patients	Ghrelin affected a circadian locomotor output cycle kaput-dependent mechanism	Garaulet et al., 2011
Abnormal eating behaviors	Healthy volunteers	Ghrelin increased appetite and food intake	Wren et al., 2001
	Male Wistar rats	Intracerebroventricular injection of ghrelin increased food intake	Wren et al., 2000
	Neuropeptide Y knockout mice	Intracerebroventricular injection of ghrelin increased food intake and body weight	Tschop et al., 2000

## Author Contributions

SK, YN, SJS, YHP, SGJ, J-iK, and MM wrote this review article. SK, YN, SJS, YHP, SGJ, J-iK, M-JK, and MM revised this review article. All authors approved the submitted version.

## Conflict of Interest

The authors declare that the research was conducted in the absence of any commercial or financial relationships that could be construed as a potential conflict of interest.

## Abbreviations

AMPK: AMP-activated protein kinase
ARC: arcuate nucleus
BDNF: brain-derived neurotrophic factor
GABA: γ-aminobutyric acid
LHA: lateral hypothalamus
NPY: neuropeptide Y
POMC: proopiomelanocortin
PVhd: dorsal parvocellular paraventricular nucleus
ROS: reactive oxygen species
SCN: suprachiasmatic nucleus
VLPO: ventrolateral preoptic nucleus.
